# An interview with Fabienne Kühne, 2024 *Epilepsia Open* prize winner for clinical research

**DOI:** 10.1002/epi4.12962

**Published:** 2024-05-23

**Authors:** Aristea S. Galanopoulou

**Affiliations:** ^1^ Saul R. Korey Department of Neurology Albert Einstein College of Medicine Bronx New York USA; ^2^ Dominick P Purpura Department of Neuroscience Albert Einstein College of Medicine Bronx New York USA

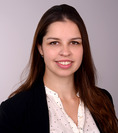

## 1 TELL US ABOUT YOURSELF

I started my academic career in Medicine, studying in Halle/Saale and in Berlin in Germany. At Charité—Universitätsmedizin Berlin, I began working in the field of pediatrics and I encountered neurological diseases for the first time professionally.

During my studies, which I finished in 2019, I started my doctoral research focusing on quantifying myelination in children using T1‐mapping in magnetic resonance imaging. This work allowed and motivated me to intensify my interest in research in pediatric neurology.

After completing my doctoral thesis, I decided to intensify my research in the field of imaging techniques to reach a higher level bytaking up a course of study in biomedical informatics, which I successfully completed. In 2023, I got my master's degree in biomedical informatics at Hochschule Mannheim, University of Applied Sciences, having worked on the application of deep neural networks in automatized ventriculometry in magnetic resonance imaging in children.

Currently, I am pursuing my pediatric residency within the field of neonatal intensive care at the Charité—Berlin University Medicine. I will complete my specialization as pediatrician by 2025.

In a part of my very limited free time, I am engaged in social projects with children through the association “Bärliner Helfen Kindern weltweit e.V. (Berliners help children worldwide)” connected with visits and stays on site in Bali, Kenya, and Cape Verde. These projects deal with children from socially disadvantaged backgrounds who should be given the opportunity to receive accommodation and education for a better future.

## 2 HOW DID YOU BECOME INTERESTED IN CONDUCTING RESEARCH IN THIS FIELD?

Angela Kaindl, Professor of Pediatrics at Charité Berlin, whose research focus is on neuropediatrics, introduced me to the highly complex and interesting research topic of epilepsy. As it is often the case, the highly specialized clinical studies cannot be transferred one‐to‐one to application in everyday clinical practice, so further extensive research is needed here.

By examining the effectiveness and safety of cannabidiol across different epilepsy subtypes, it bridges the gap between research and practical application. As we explore this avenue, we must remain vigilant, continuously refining our understanding and refining treatment approaches.

My mentor Anna Tietze, PhD., Senior physician, head of pediatric neuroradiology, gradually introduced me to research topics in the first place and got me excited about them; she continues to accompany me in this process, for which I am very grateful to her.

## 3 EXPLAIN THE QUESTION YOUR STUDY ADDRESSED, AND HOW YOU DESIGNED IT

Cannabidiol (CBD) was approved by the FDA and EMA for treating seizures in Dravet Syndrome, Lennox–Gastaut Syndrome and Tuberous Sclerosis Complex. The studies leading to approval had very narrow criteria for inclusion and exclusion of patients. In Europe, the approval for CBD requires an additional treatment with clobazam and only dosis up to 20–25 mg/kg/d are approved.

Our research group aimed to provide real‐world data for the application of CBD in epilepsy therapy. In a retrospective multicenter study, we analyzed the efficacy and tolerability of CBD in patients with epilepsy across 16 epilepsy centers. The study cohort included 311 patients with epilepsy, ranging in age from 0 to 72 years. CBD therapy was off‐label in 91.3% of cases due to factors such as age, epilepsy subtype, lack of adjunct therapy with clobazam, and higher applied doses.

## 4 WHAT WERE THE RESULTS AND HOW DO YOU INTERPRET YOUR FINDINGS?

Interpreting the findings of this study, it can be said that CBD has an antiseizure effect comparable to other antiseizure medications and has a positive safety profile, independent of the epilepsy subtype. The study also found that co‐medication with clobazam did not lead to better outcomes in seizure frequency. The safety of higher doses, particularly in children, suggests the potential for using higher doses to achieve greater reductions in seizure frequency. Furthermore, in our study data, there was no evidence to suggest a significantly better outcome for patients with the conditions for which CBD is approved compared to other patients. These results indicate the need for further trials to consider extending the approval of CBD for other epilepsy subtypes and for children under 2 years of age.

## 5 WHAT ARE THE NEXT STEPS THAT YOU PLAN TO TAKE, AND WHAT ARE YOUR CAREER GOALS?

At present, I am working on translational projects for the application of artificial intelligence for quantification and segmentation in cerebral MRI examination in children, and I am looking forward to using my newly acquired skills in biomedical informatics. As far as my next projects are concerned I would like to continue to combine clinical work, university lectures and research as well as possible.

## 6 WHAT DOES THE *EPILEPSIA OPEN* PRIZE MEAN FOR YOU, YOUR LABORATORY, RESEARCH INSTITUTE, AND YOUR FUTURE?

I am deeply honored to accept the *Epilepsia* Open Award 2024 on behalf of all the co‐authors of our research paper. For me, this award is confirmation that hard work pays off at some point and motivates me to continue my research work against all difficulties. My thanks also go to my co‐authors, who made a significant contribution to writing the research article that is now considered worthy of the award.

Receiving this award at such an early stage of my career fills me with immense pride, and I would like to express my heartfelt gratitude to Angela Kaindl for her support and conception to this project.

And, of course, I thank the patients who participated in our research—without their contributions, this project would not have been possible.

Read the prize winning article “Real‐world data on cannabidiol treatment of various epilepsy subtypes: A retrospective, multicenter study”.

## CONFLICT OF INTEREST STATEMENT

ASG is the Editor‐in‐Chief of Epilepsia Open and Associate Editor of Neurobiology of Disease. None of the authors has any conflicts to disclose in regards to this article.

## Data Availability

Data sharing not applicable to this article as no datasets were generated or analysed during the current study.

